# Medical Prescriptions

**Published:** 1870-11

**Authors:** 


### Medical Prescriptions.

The New York Legislature has recently passed an act in reference to the compounding of physicians' prescriptions. By the terms of the law no apothecary is to permit any person in his employ to put up a prescription unless said person is a medical graduate, or has served an apppenticeship of two years in a drug store. Violations of the provisions of the act are to be deemed misdemeanors, and punished by a fine of $100, or imprisonment for six months in the county jail. If death ensues, the offence is to be deemed felony, and punished by a fine of not less than $100, nor more than $500, and imprisoned in the State Prison for not less than two or more than four years, or by both fine and imprisonment, at the discretion of the court.—*Saint Louis Medical Archives.*

